# Supplementary material for: Mechanistic Insights into the Release of Doxorubicin from Graphene Oxide in Cancer Cells
Source: Nanomaterials (Basel). 2020 Jul 29;10(8):1482. doi: 10.3390/nano10081482 (PMC7466571; doi:10.3390/nano10081482)
Supplement: Supplementary file 1 [file nanomaterials-10-01482-s001.pdf]

nanomaterials

MDPI

Article

Mechanistic Insights into the Release of Doxorubicin from Graphene Oxide in Cancer Cells

Erica Quagliarini [1,†], Riccardo Di Santo [2,†], Daniela Pozzi [2,*], Paolo Tentori [3,4], Francesco Cardarelli [4] and Giulio Caracciolo [2,*]

1 Department of Chemistry, Sapienza University of Rome, P.le A. Moro 5, 00185 Rome, Italy; erica.quagliarini@uniroma1.it
2 Department of Molecular Medicine, Sapienza University of Rome, Viale Regina Elena 291, 00161 Rome, Italy; riccardo.disanto@uniroma1.it
3 Center for Nanotechnology Innovation@NEST (CNI@NEST), Istituto Italiano di Tecnologia, Piazza San Silvestro 12, 56127 Pisa, Italy; paolo.tentori@sns.it
4 NEST Laboratory, Scuola Normale Superiore, Piazza San Silvestro 12, 56127 Pisa, Italy; francesco.cardarelli@sns.it
* Correspondence: giulio.caracciolo@uniroma1.it (G.C); daniela.pozzi@uniroma1.it (D.P).
† These authors contributed equally to this work.

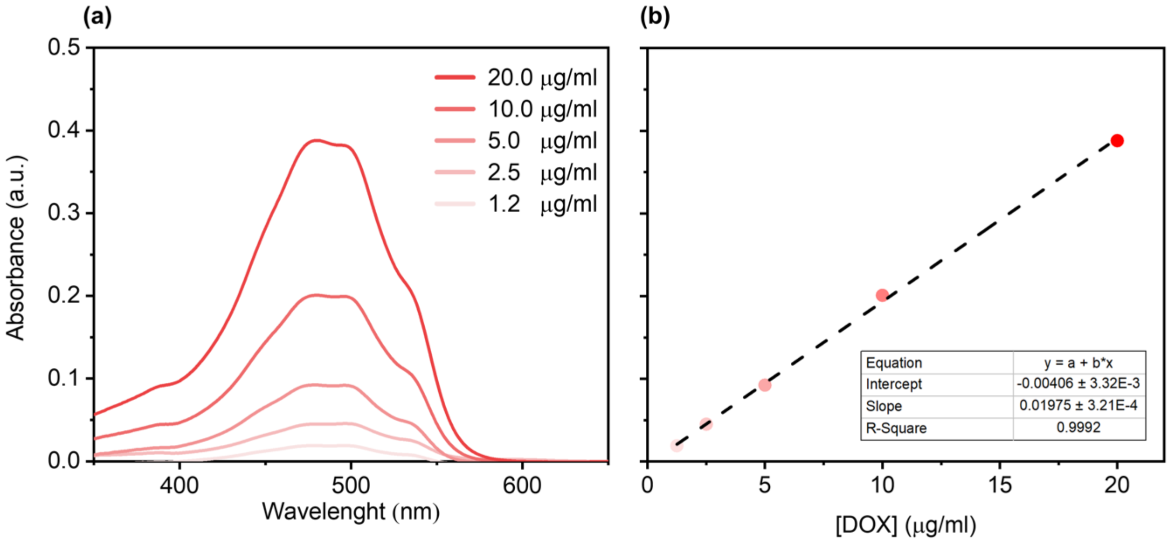

Figure S1. (**a**) Absorbance spectra of doxorubicin (DOX) at several known concentrations. (**b**) DOX calibration curve was generated from absorbance values at λ= 480 nm. The calibration curve was obtained by plotting the absorbance 480 nm versus DOX concentration and was treated by linear regression analysis (dashed line).

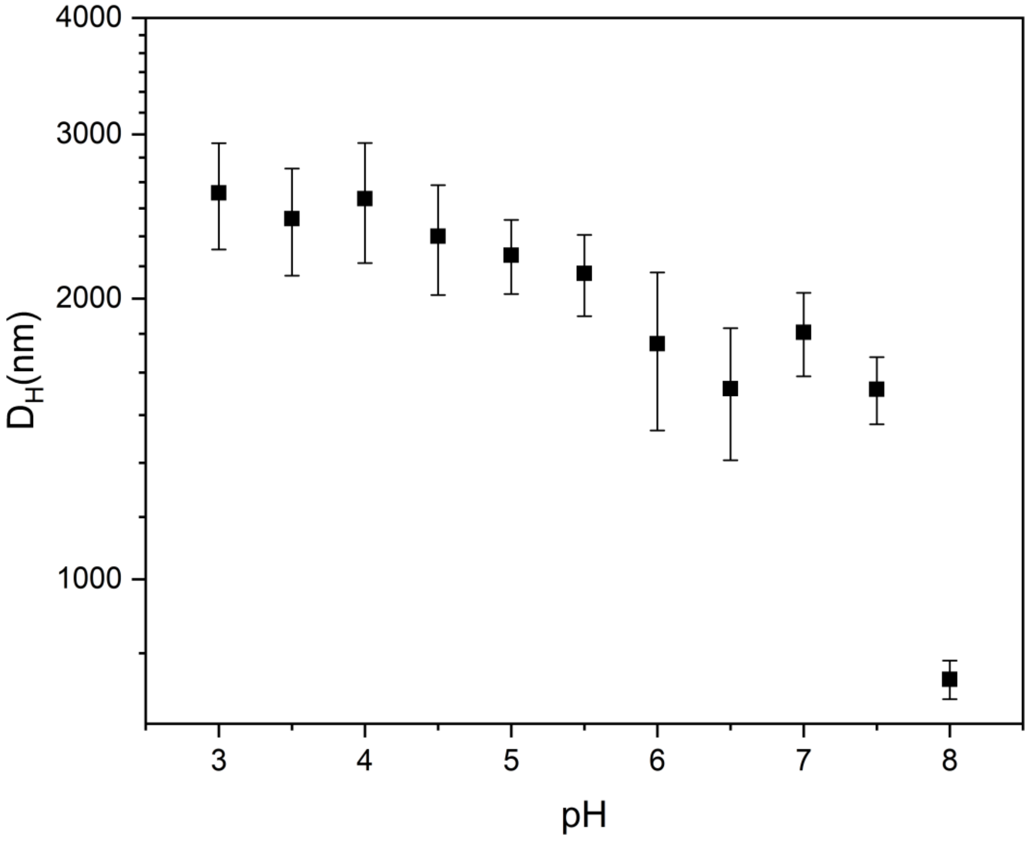

Figure S2. Hydrodinamic diameter (D_H) of GO/DOX complexes as a function of pH solution.

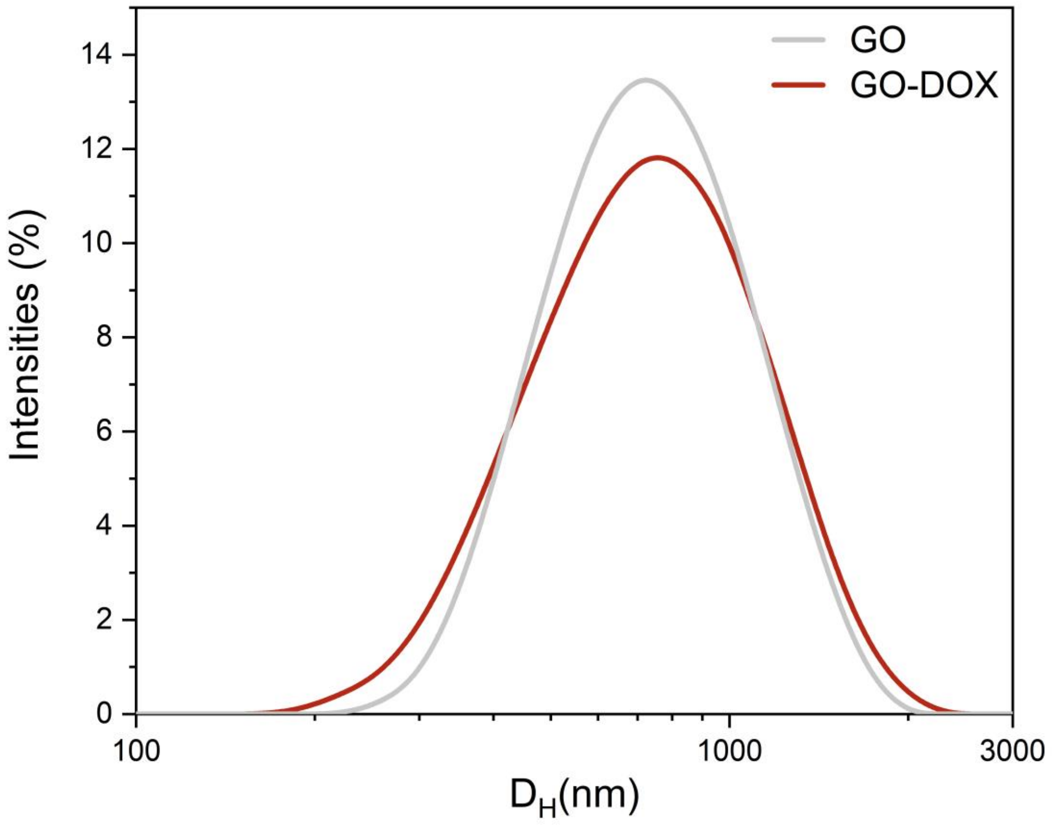

Figure S3. DH intensity-weighted distribution of pristine GO and GO/DOX complexes.

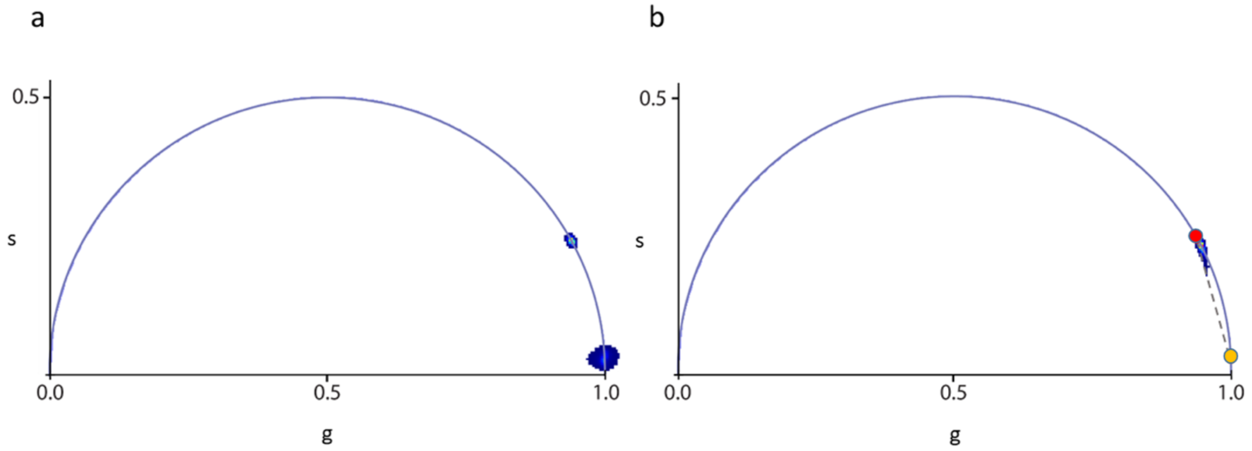

Figure S4. (**a**) The red circle highlights the experimental lifetime of DOX in aqueous solution (1 ns). The blue circle highlights the experimental lifetime of pristine graphene-oxide (GO) in aqueous solution. (**b**) The experimental cluster of GO-DOX is a linear combination of the two components, GO and DOX (represented by the red and blue dots).
